# Improved U-Net Model to Estimate Cardiac Output Based on Photoplethysmography and Arterial Pressure Waveform

**DOI:** 10.3390/s23229057

**Published:** 2023-11-09

**Authors:** Xichen Xu, Qunfeng Tang, Zhencheng Chen

**Affiliations:** 1 School of Electronic Engineering and Automation, Guilin University of Electronic Technology, Guilin 541004, China; 21082201026@mails.guet.edu.cn; 2 School of Life & Environmental Science, Guilin University of Electronic Technology, Guilin 541004, China

**Keywords:** cardiac output, photoplethysmography, arterial pressure waveform, minimally invasive, U-Net, deep learning model

## Abstract

We aimed to estimate cardiac output (CO) from photoplethysmography (PPG) and the arterial pressure waveform (ART) using a deep learning approach, which is minimally invasive, does not require patient demographic information, and is operator-independent, eliminating the need to artificially extract a feature of the waveform by implementing a traditional formula. We aimed to present an alternative to measuring cardiac output with greater accuracy for a wider range of patients. Using a publicly available dataset, we selected 543 eligible patients and divided them into test and training sets after preprocessing. The data consisted of PPG and ART waveforms containing 2048 points with the corresponding CO. We achieved an improvement based on the U-Net modeling framework and built a two-channel deep learning model to automatically extract the waveform features to estimate the CO in the dataset as the reference, acquired using the EV1000, a commercially available instrument. The model demonstrated strong consistency with the reference values on the test dataset. The mean CO was 5.01 ± 1.60 L/min and 4.98 ± 1.59 L/min for the reference value and the predicted value, respectively. The average bias was −0.04 L/min with a −1.025 and 0.944 L/min 95% limit of agreement (LOA). The bias was 0.79% with a 95% LOA between −20.4% and 18.8% when calculating the percentage of the difference from the reference. The normalized root-mean-squared error (RMSNE) was 10.0%. The Pearson correlation coefficient (r) was 0.951. The percentage error (PE) was 19.5%, being below 30%. These results surpassed the performance of traditional formula-based calculation methods, meeting clinical acceptability standards. We propose a dual-channel, improved U-Net deep learning model for estimating cardiac output, demonstrating excellent and consistent results. This method offers a superior reference method for assessing cardiac output in cases where it is unnecessary to employ specialized cardiac output measurement devices or when patients are not suitable for pulmonary-artery-catheter-based measurements, providing a viable alternative solution.

## 1. Introduction

Cardiac output (CO) refers to the total volume of blood ejected into the aorta from one side of the ventricle per minute. Cardiac output is one of the most-important indicators for assessing the health of cardiac function, and an abnormal cardiac output index may signal the onset of cardiovascular disease. By measuring cardiac output, doctors can better assess cardiac function [1]. Knowing a patient’s cardiac output before surgery can help the doctor determine the state of the patient’s heart and circulation to determine the safety and risk of the surgery. Detecting changes in cardiac output can also help the doctor assess the effectiveness of medication and whether adjustments to the treatment plan are needed.

Currently, thermodilution is considered the gold standard for measuring cardiac output, which is a method of measuring cardiac output by injecting cold saline or saline through a pulmonary artery catheter and measuring the temperature change [2,3]. However, this method can cause secondary injury to the patient and postoperative complications. Postoperative complications are the third-leading cause of death worldwide [4]. Therefore, this method is generally used only in critically ill patients and is not conducive to detecting cardiac output in general patients [5]. Our study aimed to accurately estimate cardiac output using a minimally invasive method.

Photoplethysmography (PPG) measures changes in intravascular blood flow through the skin without the need for intubation or puncture, which is non-invasive and can be performed by wearing a simple photovoltaic sensor, allowing for daily monitoring. PPG waveforms can continuously monitor changes in blood flow in real-time. These waveforms contain rich physiological information and are suitable for use in both clinical and daily monitoring settings. However, the quality of the waveform is easily affected by factors such as skin pigmentation and subcutaneous fat, and the amplitude of the waveform is relatively tiny compared with that of the arterial pressure waveform, which is easily interfered with by motion noise and other factors [6,7,8,9].

The arterial pressure waveform (ART) requires the insertion of a catheter for measurement, which is minimally invasive and less harmful to the patient than the measurement of cardiac output by pulmonary artery catheterization. The ART waveform results from the interaction of the blood pumped by the heart with the arterial system, and it contains physiological information about the heart’s function and the vascular elasticity. However, the human arterial system is very complex. The calculation process involves arterial compliance, vascular resistance, impedance, and other parameters, making estimating cardiac output challenging [10,11,12].

The percentage error is the proportion of the limit of agreement (LOA) (1.96*std) with respect to the average CO, which indicates the consistency between the two measurement methods. The larger the value, the worse the consistency is. The RMSNE is the proportion of the root-mean-squared error (RMSE) with respect to the average CO. It is an indicator used to measure the difference between the predicted value and the reference value. The smaller the value, the smaller the difference between the predicted value and the reference value is and the more accurate the estimation method is. The Pearson correlation coefficient is used to evaluate the relationship between the reference value and the estimated value. The closer the Pearson correlation coefficient is to 1, the closer the estimated value is to the reference value and the better the prediction result is. The MSE and MAE are the mean-squared error and the mean absolute error, which are used to measure the accuracy of model estimation. The smaller the value, the higher the accuracy is.

Many PPG or ART waveform studies have been conducted to predict cardiac output. Wang et al. [13] in 2009 proposed the inflection and harmonic area ratio (IHAR) of the PPG signal for CO detection, and the correlation coefficient was only 0.82, although the percentage error reached 16.2%. In 2010, Reference [14] proposed using the pulse time reflection ratio (PTRR) of ECG and PPG for estimating cardiac output, which resulted in an RMSNE of 13.15%, a percentage error of 24.90%, and a correlation coefficient of 0.87. Dean Nachman et al. [15] in 2020 evaluated the correlation between a PPG-based testing device and the Swan-Ganz invasive catheter, resulting in a value of 0.87. Ayana Dvir et al. [16] in 2022 compared the CO obtained from monitors using PPG with the CO obtained by thermodilution, resulting in a correlation coefficient of 0.906. Ke et al. [17] used arterial pressure waveforms and created a regression model to predict CO in 2022. Using the results of the random forest model, the MSE was 1.421 L/min, the 95% limit of agreement was −2.35 L/min and 2.32 L/min, the percentage error was 39.44%, being higher than 30%, and the result using the XGBoost model was 28.89%. In an article in July 2023, Alan Hamo [18] compared six models for predicting CO using arterial pressure waves. Among them, the model with the best results was the Ridge model, with a resulting MAE of 1.01 L/min and a correlation coefficient of 0.76. The percentage error was 37.96%.

Traditional methods of estimating cardiac output generally artificially extract the feature of the waveform, such as the area under the curve of the waveform [19], and then, calculate the result through a formula. Deep learning is currently being applied in different medical fields, and it has been shown that the method of estimating cardiac output using deep learningoutperforms traditional models when the blood flow is unstable [20]. Although there have been many methods for estimating cardiac output by the PPG or ART waveform, accuracy has always been an issue in cardiac output estimation. To improve the accuracy, we propose a dual-channel deep learning model based on the PPG and ART waveforms, where the PPG waveform reflects the blood flow changes in the subcutaneous vessels and the ART waveform reflects the pressure changes within the arteries. These two types of information can provide different perspectives to understand the heart’s pumping ability and circulatory state, with complementary information leading to a more-accurate estimation of cardiac output.

This study aimed to use deep learning to build a model to estimate CO using PPG waveforms and ART waveforms as the inputs. The results showed that our estimated cardiac output was highly consistent with commercial instruments, proving the feasibility of this method and providing the possibility for the realization of a more-convenient, rapid, minimally invasive, and continuous detection of cardiac output in the future.

## 2. Materials and Methods

### 2.1. Data

The VitalDB public database is a comprehensive database containing biosignal and clinical information on 6388 surgical patients. The biosignal data are high-quality data, such as waveform signals at 500 Hz and numerical signals at 1–7 s intervals. The database contains 6388 cases with 557,622 signals, measured using 11 devices [21].

The CO data collected using the commercially available instrument EV1000 (EV1000; Edwards Lifesciences, Irvine, CA, USA) in the VitalDB public database were used as the reference [22]. The PPG and ART waveforms were obtained from the analog output port of the Tram-Rac module (GE Healthcare, Chicago, IL, USA). The resolution of the ART and PPG waveforms was 500 Hz, and the CO data were digital with an interval of 2 s. There were 547 cases that contained PPG and ART waveforms and the CO. Each case intercepted the middle 60 s as the research object, specifically ((signal start time + signal end time)/2 s, (signal start time) + signal termination time)/2 + 60 s). Among the 547 data with a time period of 60 s, 4 cases had empty values. The cases with empty values were deleted, and 543 cases’ data were finally retained. The case IDs are shown in the Supplementary Material.

### 2.2. Data Preprocessing

**Figure 1:**
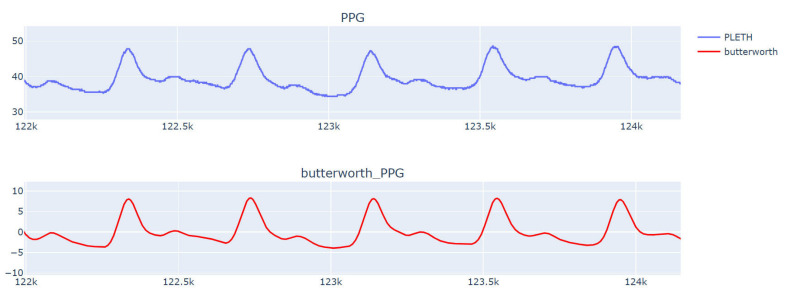
PPG original signal and filtered PPG signal.

VitalDB’s waveforms are unprocessed data due to the accelerated respiratory rate, nervousness, and accelerated heartbeat during acquisition. The waveform data contain much noise. To avoid the adverse impact of noise signals on the results, the waveform was filtered using a Butterworth second-order bandpass filter with a filtering range of 0.5–10 Hz [23]. The filtering effect is shown in Figure 1.

**Figure 2:**
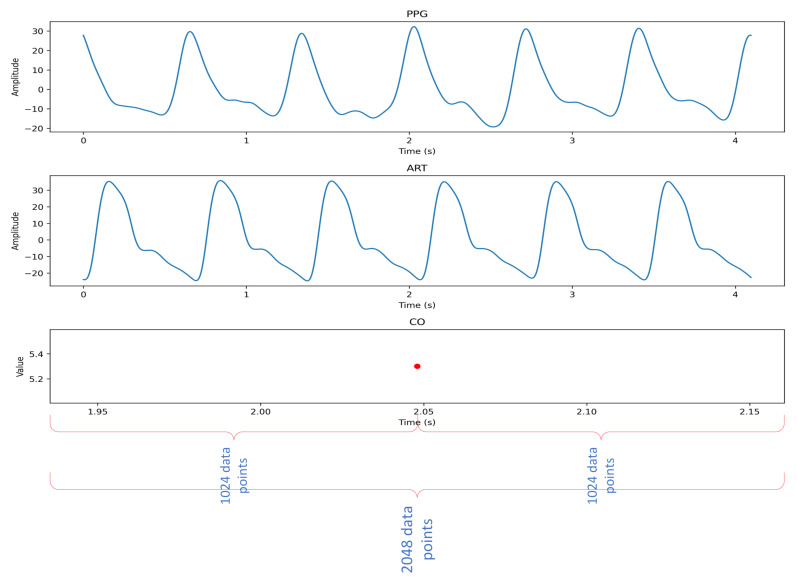
Data segments.

Generally speaking, the cardiac peak is about 1 Hz, which corresponds to 60 pulses per minute, and the respiratory peak is about 0.25 Hz, which corresponds to 15 inspiratory/expiratory cycles per minute [24]. Therefore, we needed a signal of about 4 s to obtain a complete inhalation/exhalation cycle, and the model structure determined that the number of sampling points was a multiple of 16, so we cut the PPG and ART waveforms into segments containing 2048 data points (4.096 s), the first 1024 and the last 1024 data of each of the CO data. Each PPG and ART data segment and the corresponding CO value formed a data sample, and the data segments with less than 2048 data points were deleted. A total of 14,583 data segments were obtained from 543 cases with a length of 60 s. The data segments are shown below (Figure 2).

Abnormal segments may be included in the data-acquisition process, affecting the stability and accuracy of training, so it was necessary to screen out abnormal segments. The normal value of human cardiac output is about 4.5–6 L/min. The cardiac output will be reduced when lying down and resting, and the cardiac output of some patients is less than 3.5 L/min. The cardiac output will increase during strenuous exercise, emotional excitement, etc., and may be greater than 8 L/min. Therefore, in this study, the range of the cardiac output was selected as 2 L/min to 10 L/min.

The peak value of the ART waveform is the systolic blood pressure, and the trough value is the diastolic blood pressure; the range varies according to age and individual differences. Under normal circumstances, the amplitude of the systolic blood pressure usually ranges from 90 mmHg to 140 mmHg, and the diastolic blood pressure ranges from 60 mmHg to 90 mmHg. Considering that there may be patients with low or high blood pressure in the dataset, the screening range of the ART waveform was 30 mmHg to 200 mmHg. The amplitude of the PPG waveform has no physiological significance. Still, by observing the data in the dataset, the amplitude of the standard PPG signal ranged from about 20 to 80, and considering that the signal may be low or high in exceptional cases, the screening range of the PPG waveform was 10 to 100.

The peak-to-peak value of the arterial pressure waveform is the pulse pressure, which refers to the pressure change of the artery during cardiac systole and diastole. The normal range is between 30 mmHg and 50 mmHg, so the data were considered unreliable when the range between the maximum and minimum values of the ART waveform, ARTmax-ARTmin, <30. In the PPG signals in the dataset, some of the abnormal signals were nearly straight lines. These signals had almost no helpful feature information, which affected the results of the model training, and the peak-to-peak value of the standard PPG signals was greater than 20, so if the range between the maximum and minimum values of the PPG waveforms, PPGmax-PPGmin, was <20, the segment was considered unreliable.

The resolution of the PPG and ART waveforms was 500 Hz, that is the time interval between adjacent points was 2 ms (1/500 s). We believe that, in 2 ms, from a physiological point of view, the blood flow in the blood vessels under the skin (PPG) will not change by more than 20. Similarly, the ART waveform is the intra-arterial blood pressure. Within 2 ms, the pulse pressure will not change by more than 30 [25,26]. For high-frequency noise, a large negative slope appeared in the waveform, and based on this observation, we analyzed the changes between adjacent data points. If the difference between any neighboring data points of the PPG waveform was more than 20 or the transition between neighboring data points of the ART waveform was more than 30, we marked these data as invalid, as this may be due to mutations or other anomalies.

**Figure 3:**
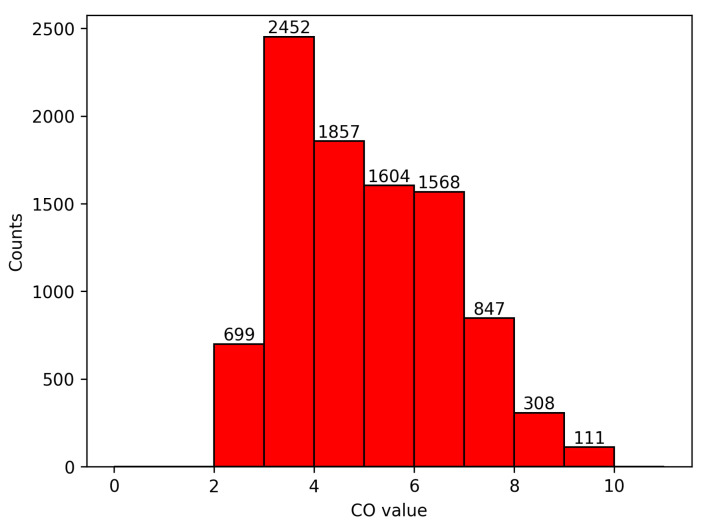
Distribution of CO values.

Through screening, we were able to eliminate outliers and noise that may affect the quality of the waveform data and increase the accuracy and stability of training. Among the 14,583 data segments, 9446 were screened out. These 9446 data segments formed the model dataset, and the average CO was 5.01 ± 1.60 L/min. The distribution of the CO is shown in Figure 3 below.

Normalization was used to linearly transform the data to between (0, 1) for a better application to deep learning.

### 2.3. Model Construction

The 9446 data samples were adjusted to the dimensions of the model inputs by matrix transformation, and the data were randomly divided into training and test sets with a division ratio of 8:2, that is 7556 data comprised the training set and 1890 data comprised the test set. In each epoch of training, 10% (756 data) was randomly removed from the training set as the validation set, and the loss of the model on unseen data was calculated. The PPG and ART waveforms in each data segment were aligned in time, that is at the same time. The segmented data were used as the input, and the CO was used as the label for model training.

In the study of non-invasive blood pressure estimation using PPG signals, U-Net performed very well, so we chose U-Net as the model architecture for this study [27,28,29]. U-Net is a convolutional neural network architecture originally proposed by Olaf Ronneberger, Philipp Fischer, and Thomas Brox in a 2015 paper [30]. It has wildly succeeded in medical image segmentation and is named after the structure’s shape, which resembles the letter “U”.

U-Net has two parts, the encoder and the decoder, which are symmetrically structured to realize the process of dimensionality reduction and the enhancement of the features. The encoder consists of multiple convolutional layers and pooling layers, which are used to gradually reduce the dimensionality of the signal and extract higher-level features, with each convolution capturing local features in the signal. The decoder also consists of multiple convolutional and upsampling layers for gradually recovering the dimensionality of the signal. Between the corresponding layers of the encoder and decoder, U-Net introduces skip connections to combine the features of the encoder with the decoded ones. The features of the device are connected to improve the accuracy. In the encoder part, the number of channels is gradually increased. In the decoder part, the number of channels is gradually reduced, which helps to extract rich features and generate the final result.

**Figure 4:**
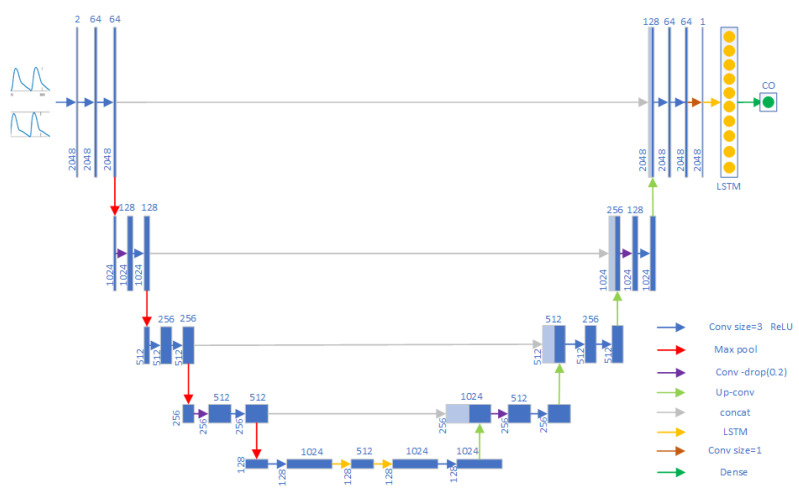
Framework diagram of the improved U-Net model.

In this study, we used a 4-layer U-Net as the model framework. U-Net is often used for two-dimensional images. In our study, the input was a one-dimensional waveform signal, and U-Net was modified to make it suitable for one-dimensional signals. The framework in this study was based on the U-Net framework by adding two layers of the bi-directional long short-term memory (LSTM) network between the decoder and encoder, as well as adding a layer of bi-directional LSTM in the output part of U-Net, then outputting the final result through the fully connected layer. LSTM is a variant of the recurrent neural network (RNN) used to process sequential data, such as time series, text, etc. Compared with the RNN, LSTM has stronger memory capability, better captures long-term dependencies, and avoids the gradient vanishing problem of the traditional RNN. The bi-directional LSTM layer combines information from both forward and reverse directions and can comprehensively capture the dependencies and features in the time series. After the experiments, the improved U-Net model showed better results than the U-Net model. The specific model framework is shown in Figure 4.

The model training environment was an RTX 3060 GPU; python was 3.9; the tensorflow GPU was 2.10. We set the model’s training parameters as follows: epochs to 500, the learning rate to 0.001, and the batch size to 32.

In the process of model training, the mean-squared error (MSE) was used as the loss function and mean absolute error (MAE) was used as the evaluation index; the formulas are shown in (1), (2), where n is the number of y.

(1) $$MSE=\frac{1}{n}\sum_{i=1}^{n}{\left(y_{i}\;-{\bar{y}}_{i}\right)}^{2}$$

(2) $$MAE=\frac{1}{n}\sum_{i=1}^{n}\left|y_{i}-{\bar{y}}_{i}\right|$$

The bias represents the average difference between the model estimate and the reference value. The closer the bias is to 0, the closer the model estimate is to the reference value. The standard deviation indicates the dispersion of the deviation, and a smaller standard deviation indicates that the deviation is concentrated near 0. The 95% LOA is used to evaluate the agreement between two measurement methods to determine whether they are substitutes for each other and is usually 1.96-times the standard deviation. The RMSE represents the average error between the model-estimated value and the reference value. The smaller the value, the closer the estimated value is to the reference value.

Due to different data and different ranges of cardiac output, comparing the LOA and RMSE may not be objective enough. For example, the deviation between measurements of 1 L/min is more significant at lower cardiac outputs than at higher cardiac outputs. In order to better compare indicators with different data ranges, the PE and RMSNE were used. The RMSNE is the ratio of the RMSE to the reference CO average, and the PE is the ratio of the LOA to the reference CO average, in percent.

The mean error (bias), standard deviation (std), 95% limit of agreement (LOA), root-mean-squared error (RMSE), root-mean-squared normalized error (RMSNE), and percent error (PE) were the evaluation indexes, and the formulas are shown in (3)–(8), where n is the number of y, $y_{p}$ is the model prediction value, and $y_{t}$ is the reference value.

(3) $$bias=\frac{1}{n}\sum_{i=1}^{n}{\left(y_{p}\;-y_{t}\right)}^{2}$$

(4) $$std=\sqrt{\frac{1}{n}\sum_{i=1}^{n}{\left[\left(y_{p}\;-y_{t}\right)-\left(y_{p}\;\overset{¯\;}{-}y_{t}\right)\right]}^{2}}$$

(5) LOA=1.96∗std

(6) $$RMSE=\sqrt{MSE}$$

(7) $$RMSNE=\frac{RMSE}{mean\left(real\;CO\right)}$$

(8) $$PE=\frac{1.96*std}{mean\left(real\;CO\right)}$$

## 3. Results

In order to evaluate whether using two signals as the input at the same time can improve the performance of the model, we conducted a comparative experiment, including the PPG waveform alone, the ART waveform alone, and the simultaneous input of the PPG and ART waveforms. The results are shown in Table 1.

By observing and comparing the experiments, we found that the results of inputting the PPG and ART waveforms at the same time were better.

**Figure 5:**
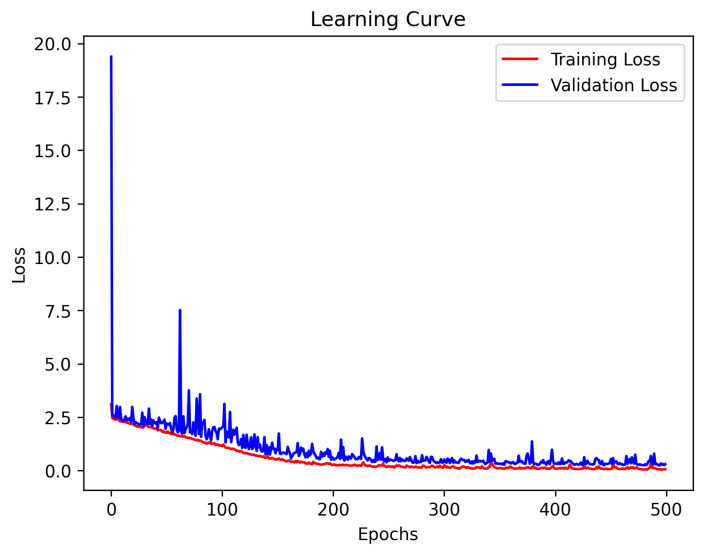
Losscurve.

In each epoch during the training process, 10% of the data were randomly divided as the validation set, and the loss value of the model on the validation set was monitored. We used the epoch with the smallest loss on the validation set as the training result. In this model, the result with Epoch 494 was selected. The loss curve is shown in Figure 5.

**Figure 6:**
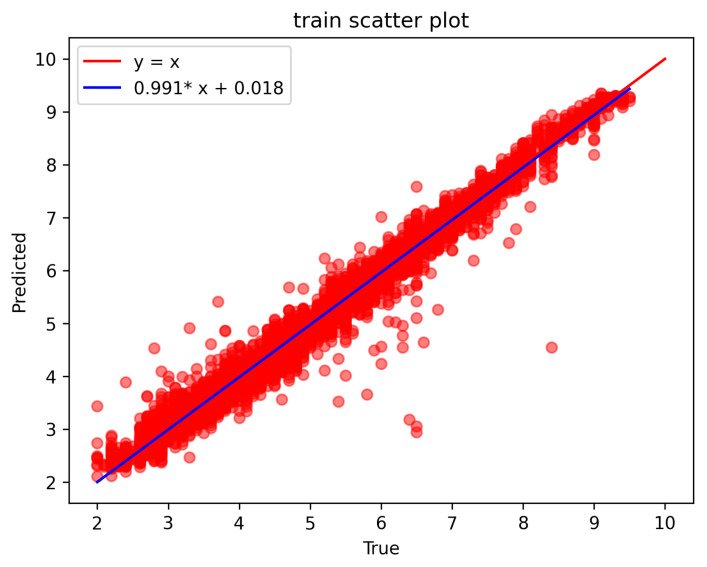
Plot of prediction results on the training set. (a) The abscissa of the red point is the reference CO, and the ordinate is the model predicted CO. (b) The blue straight line is the least squares fitting straight line of the red point y = 0.991*x + 0.018. (* represents the multiplication sign).

**Figure 7:**
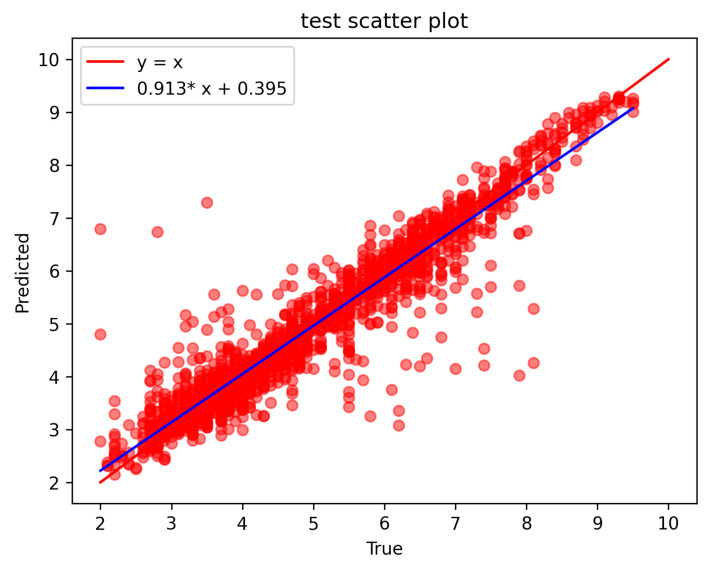
Plot of prediction results on the test set. (a) The abscissa of the red point is the reference CO, and the ordinate is the model predicted CO. (b) The blue straight line is the least squares fitting straight line of the red point y = 0.913*x + 0.395. (* represents the multiplication sign).

As shown in Figure 6 and Figure 7, the horizontal coordinate is the reference CO, the vertical coordinate is the CO predicted by the model, and the line of equivalence between the predicted value and the reference value is y = x. The blue color is the least-squares-fitted straight line to the data points, and the closer the fitted straight line is to y = x, the higher the accuracy and the better the result are. Figure 6 shows the prediction results on the training set, and Figure 7 shows the prediction results on the test set.

As can be seen from the figure, the data are evenly distributed on both sides of the equal line and are in a clustered state. The least squares fitting a straight line are very close to the equal line, indicating that the improved U-Net model’s prediction results were highly consistent with the reference value.

**Figure 8:**
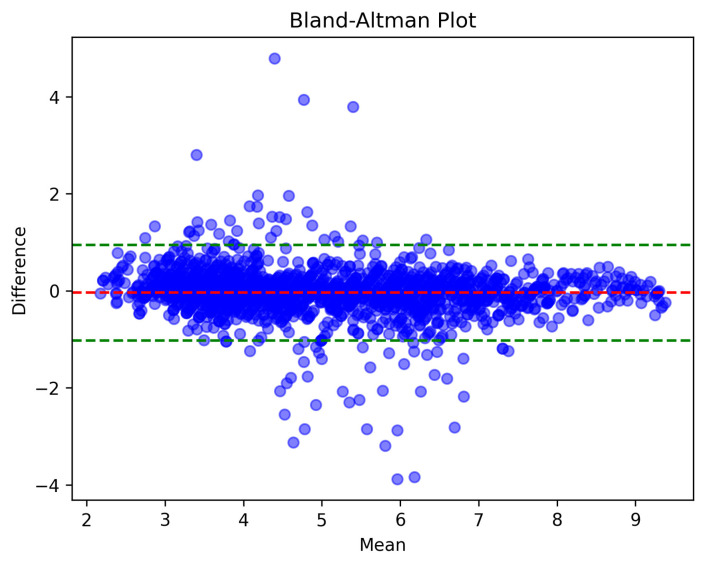
Bland–Altman plot on the test set. (a) The abscissa of the blue point is the average value of the predicted CO and the reference CO, and the ordinate is the difference between the predicted CO and the reference CO. (b) The red horizontal dashed line represents the average difference between predicted CO and reference CO, and the green horizontal dashed line represents the 95% limit of agreement.

Using the Bland–Altman plot indicated the degree of agreement between the predicted CO and the reference CO [31,32]. Figure 8 shows the Bland–Altman plot of the model on the test set, where the red horizontal dashed line represents the average difference between the predicted CO and the reference CO and the green horizontal dashed line represents the 95% limit of agreement. From the Bland–Altman plot, it can be seen that the red dashed line is close to 0, which represents a slight deviation from the model on the test set, and the narrowness between the two green dashed lines indicates that the prediction results had a small error and reasonable accuracy.

Evaluation indicators were used to indicate the quality of the results. The average deviation between the predicted results and the real results on the test set was −0.04 L/min; the standard deviation was 0.502 L/min, the 95% limit of agreement (MEAN ± 1.96 ∗ STD) was −1.025 to 0.944 L/min; the root-mean-squared error (RMSE) was 0.504 L/min; the mean-squared error (MSE) was 0.254 L/min; the mean absolute error (MAE) was 0.305 L/min; the Pearson correlation coefficient between the predicted cardiac output and true cardiac output was 0.951, as shown in Table 2.

In addition to the metrics of the correlation, mean bias, and 95% limit of agreement, the consistency of the model estimates and the reference was compared using the percentage error (PE) and the root-mean-squared normalized error (RMSNE). The RMSNE was calculated as the root-mean-squared error divided by the average true cardiac output, as shown in Equation (7), and it was 10.0% on the test set. The percentage error was calculated by dividing the accuracy by the average true cardiac output, as shown in Equation (8), and the model’s percentage error on the test set was 19.5%. This value is below the 30% error threshold proposed by L.A.H. Critchley and J.A.J.H. Critchley [33], indicating that the proposed method is clinically acceptable and outperformed the results obtained using traditional-formula-based calculations.

## 4. Discussion

The traditional use of PPG or ART waveforms to predict cardiac output is generally based on artificially extracting the features of the waveforms and combining them with conventional models, such as the Windkessel model, calculating the formula, and obtaining the cardiac output. The disadvantages are that the calculation is complex, many approximation processes lead to unsatisfactory results, and the utilization rate of the waveforms is low. It only relies on the shape of the waveform or the area under the curve and has high requirements on the quality of the waveform. Our advantage was that we used deep learning to learn the waveform features automatically and proposed a dual-channel input, that is taking the PPG and ART waveforms as the input, combining the characteristics of the two waveforms and complementary information, and achieving an end-to-end output. The results are compared as shown in Table 3.

Since the data used by Wang et al., Dean Nachman et al., and Ayana Dvir et al. were measured by the authors using instruments and Alan Hamo used the VitalDB database for comparative experiments in his research, which is the same database we used, we compared the results of Alan Hamo.

From Table 4, we improved the accuracy of our predictions by using a dual-channel input of the PPG and ART waveforms. We enhanced the U-Net model by incorporating the LSTM model, improving the overall performance. During the training, we monitored the model’s loss on unseen data and selected the optimal weights as the final result to reduce overfitting. The resulting MAE was 0.305 L/min; the percentage error was 19.5%; Pearson’s correlation coefficient was 0.951.

A PE of 30% is the clinically acceptable standard for CO monitors, but the error of most devices is 40% [16,34,35,36]. Pearson’s coefficient [37,38] and the RMSNE [39] can better represent the correlation between the reference CO and the model-estimated CO, indicating whether the method is feasible. Therefore, we paid more attention to Pearson’s coefficient and the RMSNE indicators. The results using the PE, Pearson’s correlation coefficient, and the RMSNE together generally indicated the same trends, assessing the accuracy of the estimates.

A limitation of this study was that we did not incorporate patient demographics such as gender, age, height, weight, etc. Future research may consider including this information to add individual context, potentially enhancing accuracy. Additionally, since factors like skin color and ethnicity can influence PPG measurements, so it is worth noting that the dataset used in this study was based on an open database of Asian individuals, and testing across different skin colors and ethnicities was not conducted.

During our research, we looked for publicly available datasets recording CO data. The MIMIC [40] and VitalDB public databases both contain the data required for our research, but the PPG signal of MIMIC is not the original signal, but a processed signal, which may affect the final result. After screening, there were 17 cases in the MIMIC dataset that simultaneously contained the data required for the research, which is relatively small. The sampling rate of the PPG and ABP waveforms was 125 HZ, and the sampling rate of CO was 0.9765625 Hz (not an integer); there was no alignment in time, which increased the difficulty of the research, so in this study, we only used the VitalDB dataset. We will continue to conduct in-depth research in the future.

## 5. Conclusions

Through experimentation, we discovered that using only the PPG or ART waveform had limited effectiveness. Therefore, in this study, we proposed using a dual-channel deep learning model for predicting cardiac output. With an improved U-Net model, our predicted cardiac output results closely aligned with the reference values, achieving a Pearson correlation coefficient of 0.951. It is suggested that this method is a viable alternative for CO assessment. PPG is a non-invasive measurement method, whereas recording the ART often involves minimally invasive procedures. This approach reduces harm to the human body compared to pulmonary artery catheterization. It is suitable for a broader range of patients. When specialized cardiac output measurement equipment is unavailable or patients are not ideal for pulmonary-artery-catheter-based measurements, this method can serve as an alternative, providing a reference indicator for assessing cardiac function in patients. Moreover, the PPG and ART waveforms are commonly monitored in patients. Utilizing these two waveforms for predicting the CO can lead to cost savings and increased efficiency, which is highly beneficial for ordinary patients.

## Figures and Tables

**Table 1:** Comparative experiments with different inputs.

	Bias	STD	95% LOA	MAE	MSE	RMSE	RMSNE		
Input	(**L/min**)	**(L/min)**	**(L/min)**	**(L/min)**	**(L/min)**	**(L/min)**	**(L/min)**	R	PE
PPG	−0.001	1.564	−3.067 to 3.065	1.295	2.447	1.564	31.10%	0.27	60.90%
ART	−0.04	0.631	−1.277 to 1.199	0.398	0.4	0.633	12.60%	0.921	24.60%
PPG and ART	−0.04	0.502	−1.025 to 0.944	0.305	0.254	0.504	10.00%	0.951	19.50%

**Table 2:** Evaluation metrics of the model for the test set.

Mean deviation (MEAN)	−0.04 L/min
Standard deviation (STD)	0.502 L/min
95% limit of agreement (LOA)	−1.025 0.944 L/min
RMSE	0.504 L/min
RMSNE	10.00%
PE	19.50%
MSE	0.254 L/min
MAE	0.305 L/min
Pearson’s correlation coefficient (r)	0.951

**Table 3:** Paper comparison.

Author	Input	Model	Bias (L/min)	95% LOA (L/min)	MSE (L/min)	RMSNE	r	PE
Wang et al. [13]	PPG	The inflection and harmonic area ratio	NR	1.4	NR	NR	0.82	16.20%
	ECG, PPG	Calibration I	−0.06	1.97	NR	11.79%	0.88	23.43%
	ECG, PPG	Calibration II	−0.22	2.12	NR	13.15%	0.87	24.90%
Dean Nachman et al. [15]	PPG	Testing equipment	NR	NR	NR	NR	0.87	NR
Ayana Dvir et al. [16]	PPG	PPG-based monitors	0.3	1.9	NR	NR	0.906	NR
Ke et al. [17]	ART	Random forest model	−0.01	2.33	1.421	NR	NR	39.44%
	ART	XGBoost model	NR	NR	NR	NR	NR	28.89%
Alan Hamo [18]	ART	Ridge model	−0.04	2.24	NR	NR	0.6	44.77%
This paper	PPG, ART	Improved U-Net model	−0.04	0.984	0.254	10.00%	0.951	19.50%

**Table 4:** Comparison under the VitalDB database.

Author	Bias (L/min)	95% LOA (L/min)	MAE (L/min)	R	PE
Alan Hamo [18]	−0.07	−2.23 to 2.10	0.94	0.82	33.34%
This paper	−0.04	−1.025 to 0.944	0.305	0.951	19.50%

## Data Availability

The data presented in this study are openly available in VitalDB at DOI: 10.1038/s41597-022-01411-5 [21].

## Supplementary Materials

The following supporting information can be downloaded at: https://www.mdpi.com/article/10.3390/s23229057/s1.

## Abbreviations

The following abbreviations are used in this manuscript:

CO	Cardiac output
PPG	Photoplethysmography
ABP	Arterial blood pressure (invasive, from one of the radial arteries)
ART	Arterial blood pressure (invasive, from the other radial artery)
ECG	Electrocardiogram

## References

[1] Pinsky M.R. (2003). Why measure cardiac output?. Crit. Care.

[2] Drummond K.E., Murphy E. (2012). Minimally invasive cardiac output monitors. Contin. Educ. Anaesthesia Crit. Care Pain.

[3] Marik P.E. (2013). Obituary: Pulmonary artery catheter 1970 to 2013. Ann. Intensive Care.

[4] Nepogodiev D., Martin J., Biccard B., Makupe A., Bhangu A., Ademuyiwa A., Adisa A.O., Aguilera M.L., Chakrabortee S., Fitzgerald J.E. (2019). Global burden of postoperative death. Lancet.

[5] Buhre W., Rossaint R. (2003). Perioperative management and monitoring in anaesthesia. Lancet.

[6] Lee Q.Y., Redmond S.J., Chan G.S., Middleton P.M., Steel E., Malouf P., Critoph C., Flynn G., O’Lone E., Lovell N.H. (2013). Estimation of cardiac output and systemic vascular resistance using a multivariate regression model with features selected from the finger photoplethysmogram and routine cardiovascular measurements. Biomed. Eng. Online.

[7] Elgendi M. (2012). On the analysis of fingertip photoplethysmogram signals. Curr. Cardiol. Rev..

[8] Wang C., Huang C., Ye S. Noninvasive cardiac output monitoring system based on photoplethysmography. Proceedings of the 2014 IEEE International Conference on Progress in Informatics and Computing.

[9] McCombie D.B., Reisner A.T., Asada H.H. (2005). Laguerre-model blind system identification: Cardiovascular dynamics estimated from multiple peripheral circulatory signals. IEEE Trans. Biomed. Eng..

[10] Deng Z., Zhang C., Yu P., Shao J., Liang F. (2014). Estimation of left ventricular stroke volume based on pressure waves measured at the wrist: A method aimed at home-based use. Bio-Med. Mater. Eng..

[11] Saugel B., Cecconi M., Wagner J., Reuter D. (2015). Noninvasive continuous cardiac output monitoring in perioperative and intensive care medicine. Br. J. Anaesth..

[12] Mansencal N., Delobelle J., Balagny P., Badie J., Ihaddaden M., Arslan M., Dubourg O. (2013). Usefulness of a noninvasive cardiac output measurement using pulse wave transit time in coronary care unit. Int. J. Cardiol..

[13] Wang L., Pickwell-MacPherson E., Liang Y., Zhang Y.T. Noninvasive cardiac output estimation using a novel photoplethysmogram index. Proceedings of the 2009 Annual International Conference of the IEEE Engineering in Medicine and Biology Society.

[14] Wang L., Poon C., Zhang Y. (2010). The non-invasive and continuous estimation of cardiac output using a photoplethysmogram and electrocardiogram during incremental exercise. Physiol. Meas..

[15] Nachman D., Constantini K., Poris G., Wagnert-Avraham L., Gertz S.D., Littman R., Kabakov E., Eisenkraft A., Gepner Y. (2020). Wireless, non-invasive, wearable device for continuous remote monitoring of hemodynamic parameters in a swine model of controlled hemorrhagic shock. Sci. Rep..

[16] Dvir A., Goldstein N., Rapoport A., Balmor R.G., Nachman D., Merin R., Fons M., Ishay A.B., Eisenkraft A. (2022). Comparing Cardiac Output Measurements Using a Wearable, Wireless, Noninvasive Photoplethysmography-Based Device to Pulse Contour Cardiac Output in the General ICU: A Brief Report. Crit. Care Explor..

[17] Ke L., Elibol A., Wei X., Cenyu L., Wei W., Chong N.Y. Machine Learning Algorithm to Predict Cardiac Output Using Arterial Pressure Waveform Analysis. Proceedings of the 2022 IEEE International Conference on Bioinformatics and Biomedicine (BIBM).

[18] Hamo A. (2023). Machine Learning Algorithm to Estimate Cardiac Output Based on Non-Invasive Arterial Blood Pressure Measurements. Master’s Thesis.

[19] Daimiwal N., Sundhararajan M. (2016). Non invasive measurement and analysis of cardiac output for different age group using ppg sensor. Int. J. Comput. Appl..

[20] Moon Y.J., Moon H.S., Kim D.S., Kim J.M., Lee J.K., Shim W.H., Kim S.H., Hwang G.S., Choi J.S. (2019). Deep learning-based stroke volume estimation outperforms conventional arterial contour method in patients with hemodynamic instability. J. Clin. Med..

[21] Lee H.C., Park Y., Yoon S.B., Yang S.M., Park D., Jung C.W. (2022). VitalDB, a high-fidelity multi-parameter vital signs database in surgical patients. Sci. Data.

[22] Park M., Han S., Kim G.S., Gwak M.S. (2016). Evaluation of new calibrated pulse-wave analysis (VolumeViewTM/EV1000TM) for cardiac output monitoring undergoing living donor liver transplantation. PLoS ONE.

[23] Zhang X., Jiang S. Application of fourier transform and butterworth filter in signal denoising. Proceedings of the 2021 6th International Conference on Intelligent Computing and Signal Processing (ICSP).

[24] Daimiwal N., Sundhararajan M., Shriram R. Respiratory rate, heart rate and continuous measurement of BP using PPG. Proceedings of the 2014 International Conference on Communication and Signal Processing.

[25] Sun J., Reisner A., Mark R. A signal abnormality index for arterial blood pressure waveforms. Proceedings of the 2006 Computers in Cardiology.

[26] Li Q., Clifford G.D. Suppress false Arrhythmia alarms of ICU monitors using heart rate estimation based on combined arterial blood pressure and ECG analysis. Proceedings of the 2008 2nd International Conference on Bioinformatics and Biomedical Engineering.

[27] Athaya T., Choi S. (2021). An estimation method of continuous non-invasive arterial blood pressure waveform using photoplethysmography: A U-Net architecture-based approach. Sensors.

[28] Mahmud S., Ibtehaz N., Khandakar A., Tahir A.M., Rahman T., Islam K.R., Hossain M.S., Rahman M.S., Musharavati F., Ayari M.A. (2022). A shallow U-Net architecture for reliably predicting blood pressure (BP) from photoplethysmogram (PPG) and electrocardiogram (ECG) signals. Sensors.

[29] Athaya T., Choi S. (2022). Real-Time Cuffless Continuous Blood Pressure Estimation Using 1D Squeeze U-Net Model: A Progress toward mHealth. Biosensors.

[30] Ronneberger O., Fischer P., Brox T. (2015). U-net: Convolutional networks for biomedical image segmentation. Medical Image Computing and Computer-Assisted Intervention–MICCAI 2015, Proceedings of the 18th International Conference, Munich, Germany, 5–9 October 2015.

[31] Bland J.M., Altman D. (1986). Statistical methods for assessing agreement between two methods of clinical measurement. Lancet.

[32] Bland J.M., Altman D.G. (1999). Measuring agreement in method comparison studies. Stat. Methods Med. Res..

[33] Critchley L.A., Critchley J.A. (1999). A meta-analysis of studies using bias and precision statistics to compare cardiac output measurement techniques. J. Clin. Monit. Comput..

[34] Peyton P.J., Chong S.W. (2010). Minimally invasive measurement of cardiac output during surgery and critical care: A meta-analysis of accuracy and precision. J. Am. Soc. Anesthesiol..

[35] Peyton P.J., Chong S.W. (2011). Bias and precision statistics: Should we still adhere to the 30% benchmark for cardiac output monitor validation studies?. J. Am. Soc. Anesthesiol..

[36] Cecconi M., Rhodes A., Poloniecki J., Della Rocca G., Grounds R.M. (2009). Bench-to-bedside review: The importance of the precision of the reference technique in method comparison studies–with specific reference to the measurement of cardiac output. Crit. Care.

[37] Pearson K. (1909). Determination of the coefficient of correlation. Science.

[38] Palmers P.J., Vidts W., Ameloot K., Cordemans C., Van Regenmortel N., De Laet I., Schoonheydt K., Dits H., Eichhorn V., Reuter D. (2012). Assessment of three minimally invasive continuous cardiac output measurement methods in critically ill patients and a review of the literature. Anaesthesiol. Intensive Ther..

[39] Chen T., Clifford G., Mark R. The effect of signal quality on six cardiac output estimators. Proceedings of the 2009 36th Annual Computers in Cardiology Conference (CinC).

[40] Moody G.B., Mark R.G. A database to support development and evaluation of intelligent intensive care monitoring. Proceedings of the Computers in Cardiology.

